# Variations of subtelomeric tandem repeats and rDNA on chromosome 1RS arms in the genus *Secale* and 1BL.1RS translocations

**DOI:** 10.1186/s12870-022-03598-6

**Published:** 2022-04-25

**Authors:** Jie Luo, Ruiying Liao, Yanling Duan, Shulan Fu, Zongxiang Tang

**Affiliations:** 1 College of Agronomy, Sichuan Agricultural University, Wenjiang, Chengdu, 611130 Sichuan China; 2 Provincial Key Laboratory for Plant Genetics and Breeding, Wenjiang, Chengdu, 611130 Sichuan China

**Keywords:** Wheat, Rye, 1BL.1RS, 1RS polymorphism, Tandem repeat

## Abstract

**Background:**

The wheat-rye 1BL.1RS translocations have played an important role in common wheat breeding programs. Subtelomeric tandem repeats have been often used to investigate polymorphisms of 1RS arms, but further research about their organizations on the 1RS chromosome is needed.

**Results:**

162 1RS arms from a wild rye species (*Secale strictum*) and six cultivated rye accessions (*Secale cereale* L.) (81 plants), 102 1BL.1RS and one 1AL.1RS translocations were investigated using oligo probes Oligo-TaiI, Oligo-pSc119.2–1, Oligo-pTa71A-2, Oligo-pSc200 and Oligo-pSc250, which were derived from tandem repeats *Tai*I, pSc119.2, pTa71, pSc200 and pSc250, respectively. The variations of 1RS arms were revealed by signal intensity of probes Oligo-pSc119.2–1, Oligo-pTa71A-2, Oligo-pSc200 and Oligo-pSc250. Proliferation of rDNA sequences on the 1RS chromosomes was observed. According to the presence of probe signals, 34, 127 and 144 of the 162 1RS arms contained *Tai*I, pSc200 and pSc250, respectively, and all of them contained pSc119.2 and pTa71. Most of the 1RS arms in rye contained three kinds of subtelomeric tandem repeats, the combination of pSc119.2, pSc200 and pSc250 was most common, and only eight of them contained *Tai*I, pSc119.2, pSc200 and pSc250. All of the 1RS arms in 1BL.1RS and 1AL.1RS translocations contained pSc119.2, pTa71, pSc200 and pSc250, but the presence of the *Tai*I family was not observed.

**Conclusion:**

New organizations of subtelomeric tandem repeats on 1RS were found, and they reflected new genetic variations of 1RS arms. These 1RS arms might contain abundant allelic diversity for agricultural traits. The narrow genetic base of 1RS arms in 1BL.1RS and 1AL.1RS translocations currently used in agriculture is seriously restricting their use in wheat breeding programs. This research has found new 1RS sources for the future restructuring of 1BL.1RS translocations. The allelic variations of these 1RS arms should be studied more intensely as they may enrich the genetic diversity of 1BL.1RS translocations.

**Supplementary Information:**

The online version contains supplementary material available at 10.1186/s12870-022-03598-6.

## Background

Cultivars carrying the wheat-rye 1BL.1RS centric fusion chromosomes have been used extensively around the world in commercial agriculture. Thirty-five of the 66 (53%) Hungarian-bred wheat varieties registered in Hungary between 1978 and 1999 carried the 1BL.1RS translocations [1]. In Bulgaria, 54% of wheat cultivars contained 1BL.1RS translocations [2]. In China, 38% of wheat cultivars released since 1980 contained 1BL.1RS translocations [3]. These previous reports confirm that 1BL.1RS translocation chromosomes have played an important role in wheat breeding programs. Although some important disease resistance genes on 1RS are no longer effective, recent studies show that 1BL.1RS translocation chromosomes are still being used widely in Chinese wheat cultivars [4–6] because they are associated with beneficial effects on grain yield and improved tolerance under abiotic stress [7–10]. However, the 1RS chromosome arm in these cultivars was derived from just a single genotype cv. Petkus (*S. cereale* L.) [11]. The root biomass and responses to drought were assessed on six 1BL.1RS translocations in which 1RS arms were from different sources, and no significant differences were observed among them [12]. Therefore, to exploration of new 1RS sources for use in commercial cultivars is a high research priority for wheat geneticists.

1RS-specific markers have often been used to investigate the polymorphisms of rye cultivars (*Secale cereale* L.) and 1BL.1RS translocations [13–18]. These markers have successfully distinguished between the 1AL.1RS and 1BL.1RS translocations where the 1RS segments were derived from rye cultivars Insave and Petkus, respectively [16, 18]. However, in 78 other wheat-rye 1BL.1RS translocation cultivars/lines, the diversity of the 1RS segments was not obvious using current 1RS-specific markers [15]. Furthermore, high genetic conservation between the 1RS segments of *S. africanum* Stapf. and *S. cereale* L. was also observed using 1RS-specific markers [17]. In contrast, diversity of these 1RS segments was obvious using fluorescence in situ hybridization (FISH) analyses that are also useful for displaying chromosome architecture [19]. The subtelomeric regions of rye 1R chromosomes carry several nonhomologous tandem repeats including pSc119.2, pSc200, pSc250 and *Tai*I family [20, 21]. All these tandem repeats belong to subtelomeric satellites [22]. The structural variations of 1RS from different *Secale* accessions have already been detected by FISH analysis using pSc119.2, pSc200 and pSc250 as probes [23, 24]. Nevertheless, these tandem repeats were not used simultaneously to study the structure of 1RS arms [23, 24], and the information about the organization of subtelomeric tandem repeats on 1RS is still limited. The *Tai*I family was derived from *Leymus racemosus* and exists widely in the Triticeae tribe [25]. Although it was reported that the *Tai*I family is present on the 1RS arm [21], its variation is unknown. The 1RS arm also contains a nucleolus organizer region (NOR) with tandem repeats. Studies into the complexity of the organization of tandem repeats on 1RS can provide background information relating to the use of 1RS in wheat breeding programs. Additionally, it is unclear whether there are structural alterations of subtelomeric regions among the 1RS chromosome segments that have been successfully used in wheat cultivars/lines. In this study, oligonucleotide (oligo) probes derived from tandem repeats pSc119.2 [26], pSc200, pSc250 [20], the *Tai*I family [25] and pTa71 with NOR repeats [27] were used to investigate the structure of 1RS arms in the 1BL.1RS translocations, wild rye (*S. strictum*) and cultivated rye (*S. cereale* L.). The purpose of this study is to uncover the extent of polymorphisms of the 1BL.1RS chromosomes in commercial wheat cultivars and to explore new 1RS sources for wheat cultivar improvement.

## Results

### Organization of tandem repeats on 1RS arms in rye

**Fig. 1:**
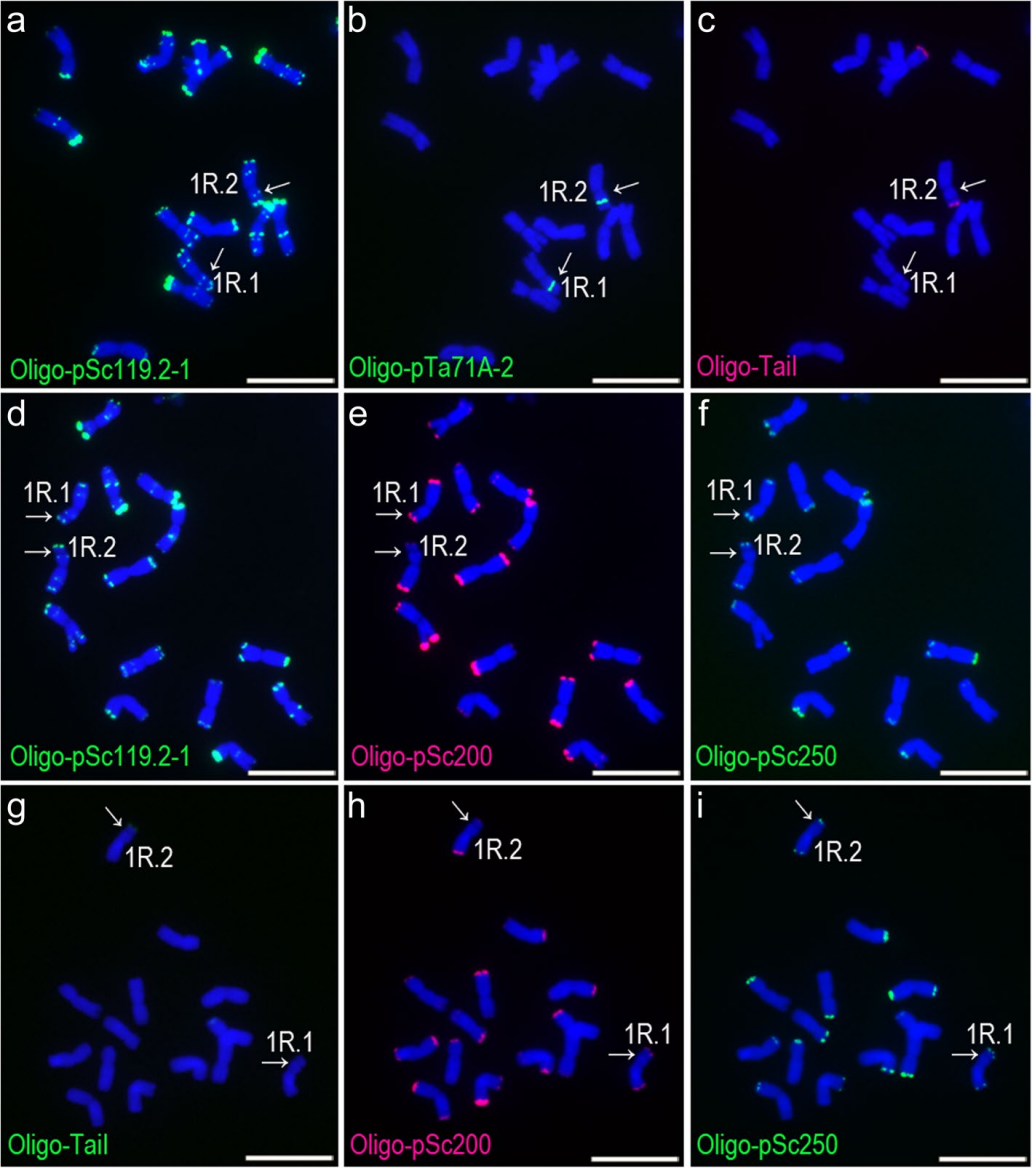
ND-FISH analysis of three root-tip metaphase cells of rye plant PI 330,965–7. **a**, **b**, **c** A cell hybridized with Oligo-pSc119.2, Oligo-pTa71A-2 and Oligo-TaiI. **d**, **e**, **f** A second cell hybridized with Oligo-pSc119.2, Oligo-pSc200 and Oligo-pSc250. **g**, **h**, **i** A third cell hybridized with Oligo-TaiI, Oligo-pSc200 and Oligo-pSc250. The 1RS of 1R.1 and 1R.2 represent types TaiI^N^-200^C^-250^C^ and TaiI^C^-200^C^-250^C^, respectively. The arrows indicate 1RS arms. Scale bar: 10 μm

**Fig. 2:**
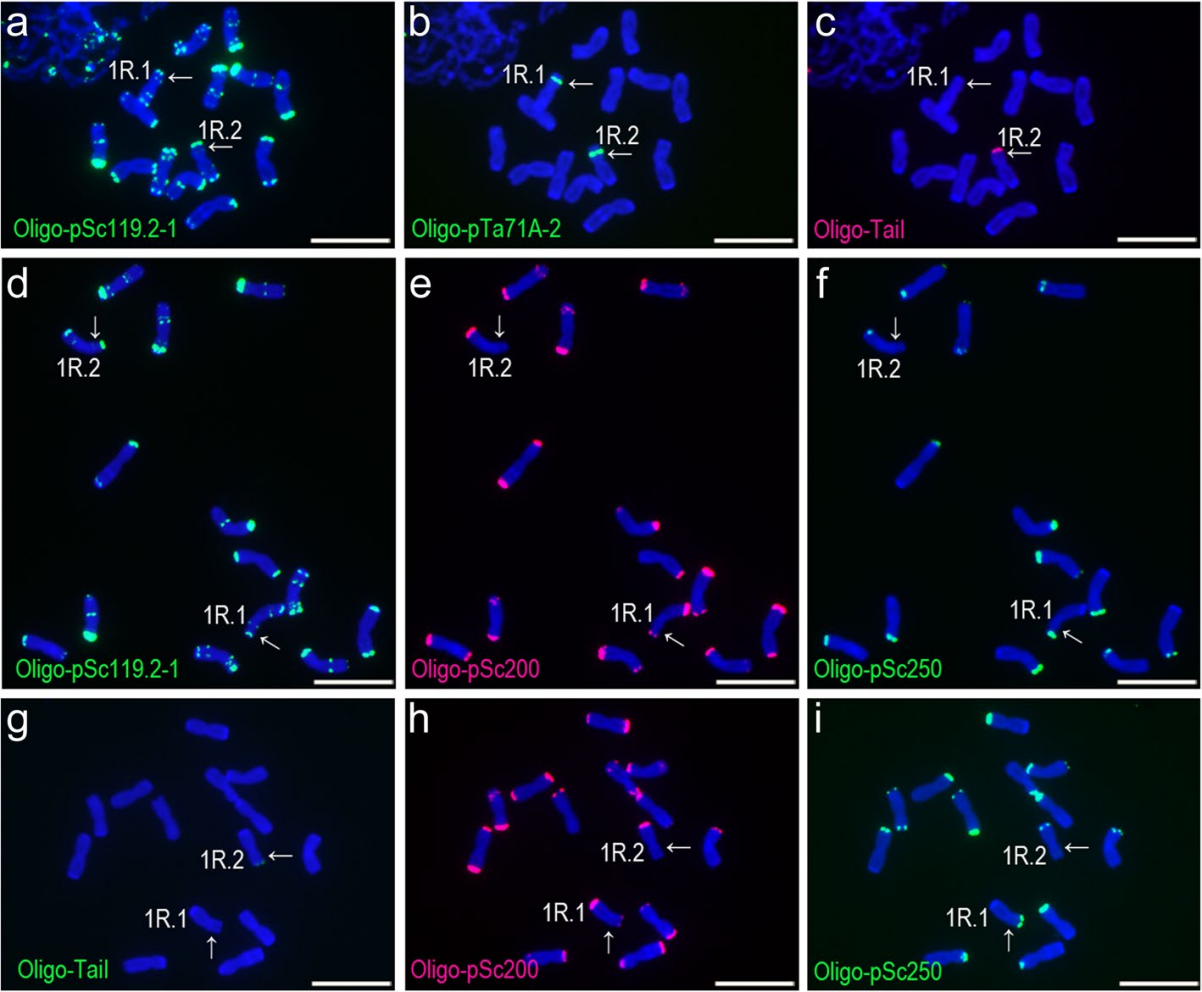
ND-FISH analysis of three root-tip metaphase cells of rye plant PI 535,171–2. **a**, **b**, **c** A cell hybridized with Oligo-pSc119.2, Oligo-pTa71A-2 and Oligo-TaiI. **d**, **e**, **f** A second cell hybridized with Oligo-pSc119.2, Oligo-pSc200 and Oligo-pSc250. **g**, **h**, **i** A third cell hybridized with Oligo-TaiI, Oligo-pSc200 and Oligo-pSc250. The 1RS of 1R.1 and 1R.2 represent types TaiI^N^-200^C^-250^C^ and TaiI^C^-200^ N^-250^ N^, respectively. The arrows indicate 1RS arms. Scale bar: 10 μm

**Fig. 3:**
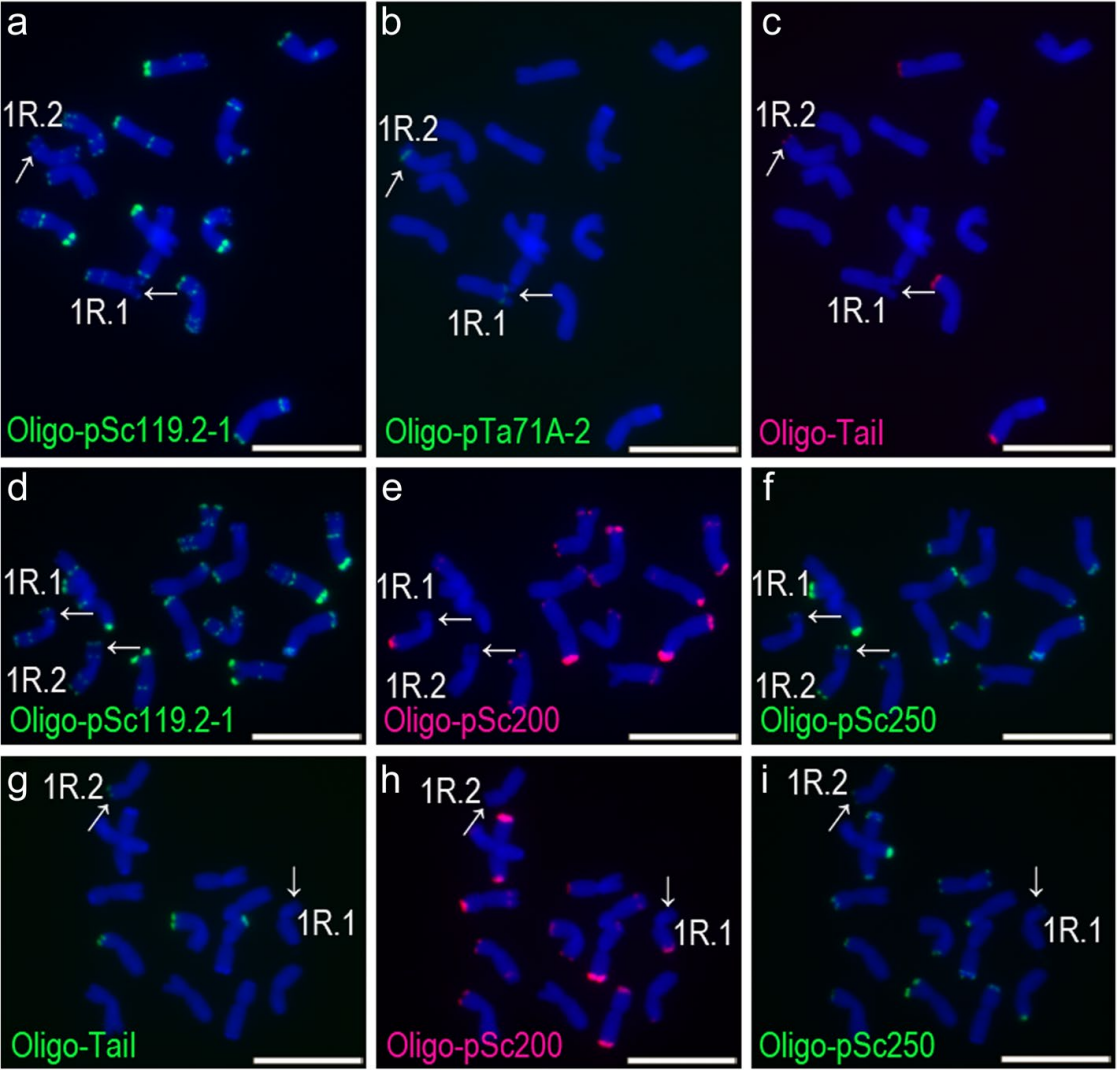
ND-FISH analysis of three root-tip metaphase cells of rye plant PI 446,244–11. **a**, **b**, **c** A cell hybridized with Oligo-pSc119.2, Oligo-pTa71A-2 and Oligo-TaiI. **d**, **e**, **f** A second cell hybridized with Oligo-pSc119.2, Oligo-pSc200 and Oligo-pSc250. **g**, **h**, **i** A third cell hybridized with Oligo-TaiI, Oligo-pSc200 and Oligo-pSc250. The 1RS of 1R.1 and 1R.2 represent types TaiI^N^-200^ N^-250^C^ and TaiI^C^-200^ N^-250^C^, respectively. The arrows indicate 1RS arms. Scale bar: 10 μm

**Fig. 4:**
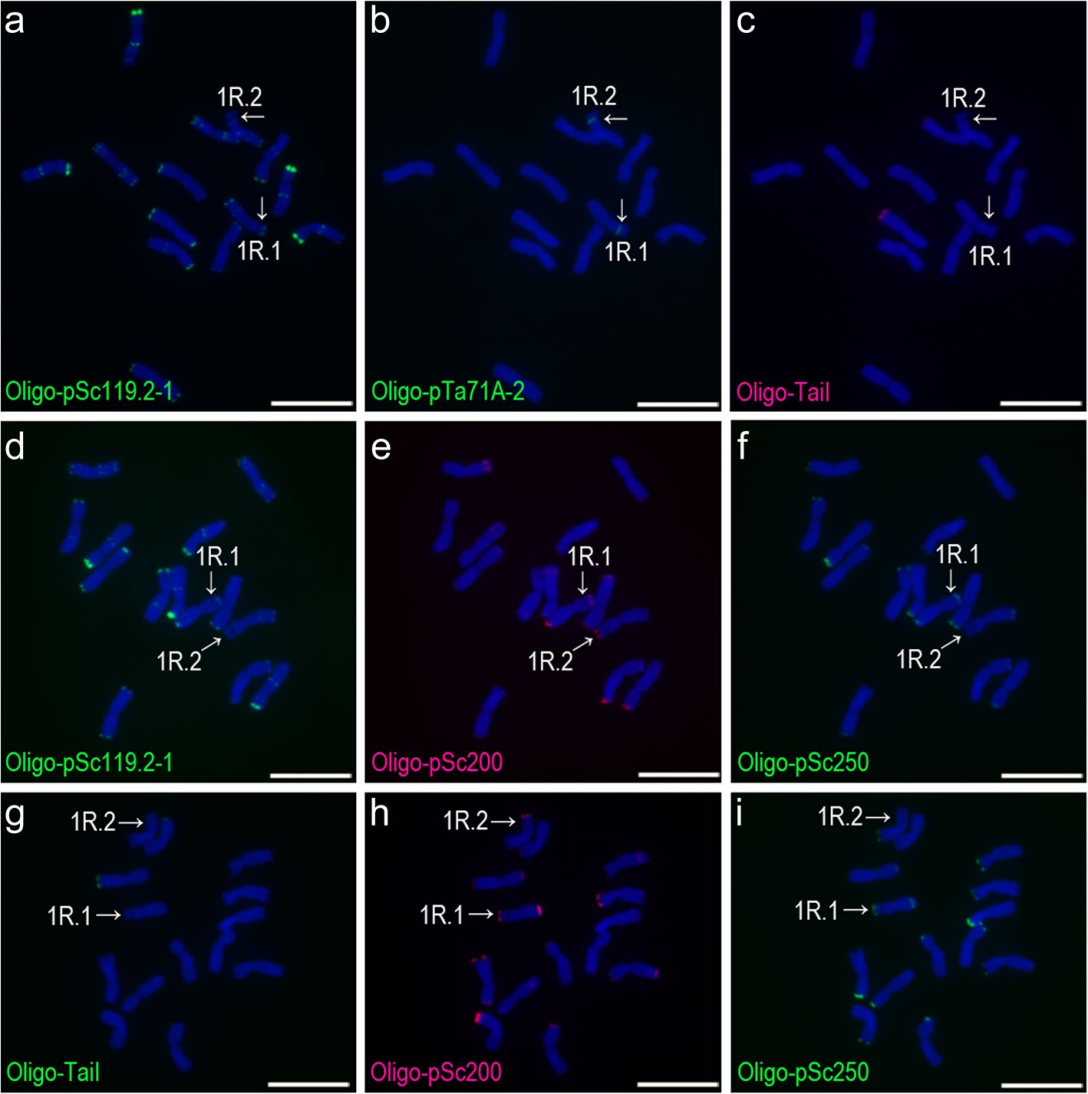
ND-FISH analysis of three root-tip metaphase cell of rye plant Jinzhou-4. **a**, **b**, **c** The cell hybridized with Oligo-pSc119.2, Oligo-pTa71A-2 and Oligo-TaiI. **d**, **e**, **f** Another cell hybridized with Oligo-pSc119.2, Oligo-pSc200 and Oligo-pSc250. **g**, **h**, **i** A different cell hybridized with Oligo-TaiI, Oligo-pSc200 and Oligo-pSc250. The 1RS of 1R.1 and 1R.2 represent types TaiI^N^-200^C^-250^C^ and TaiI^N^-200^C^-250^ N^, respectively. The arrows indicate 1RS arms. Scale bar: 10 μm

Eleven plants from PI 315,959 (Petkus Kurzstroh) and 20 plants from PI 392,065 (Kustro) were analyzed. For each of the other five accessions of rye, we worked with ten plants. A total of 162 1RS arms (from 81 plants) were studied. In order to avoid errors caused by different probe concentrations and image exposure times, the different FISH karyotypes among the 162 sources of 1RS were distinguished by signal presence or absence rather than signal intensity of probes. Probes Oligo-pTa71A-2 and Oligo-pSc119.2–1 produced signals on all of the 162 1RS arms, while the signals of Oligo-TaiI, Oligo-pSc200 and Oligo-pSc250 were absent from some 1RS chromosomes (Figs. 1, 2, 3 and 4, Additional files 1, 2, 3, 4, 5, 6 and 7). Therefore, according to the signal patterns of Oligo-TaiI, Oligo-pSc200 and Oligo-pSc250, six FISH karyotypes including TaiI^N^-200^C^-250^C^, TaiI^C^-200^ N^-250^ N^, TaiI^C^-200^ N^-250^C^, TaiI^N^-200^ N^-250^C^, TaiI^C^-200^C^-250^C^ and TaiI^N^-200^C^-250^ N^ were observed among the 162 1RS chromosomes (Figs. 1, 2, 3 and 4, Additional file 8). Type TaiI^N^-200^C^-250^C^ indicates a 1RS chromosome with both pSc200 and pSc250, and without *Tai*I. Type TaiI^C^-200^ N^-250^ N^ indicates a 1RS arm with *Tai*I but without pSc200 and pSc250, and so on. Only eight 1RS arms contained all of the four kinds of subtelomeric tandem repeats. 116 of the 1RS arms contained pSc119.2, pSc200 and pSc250, and 11 of the 1RS arms contained pSc119.2, *Tai*I and pSc250. Furthermore, 15, nine and three 1RS arms contained pSc119.2 and *Tai*I, pSc119.2 and pSc250, and pSc119.2 and pSc200, respectively (Additional file 8). Obviously, most of the 1RS in wild and cultivated rye accessions contained three kinds of subtelomeric tandem repeats, and the combination of pSc119.2, pSc200 and pSc250 was most common (Additional file 8).

**Fig. 5:**
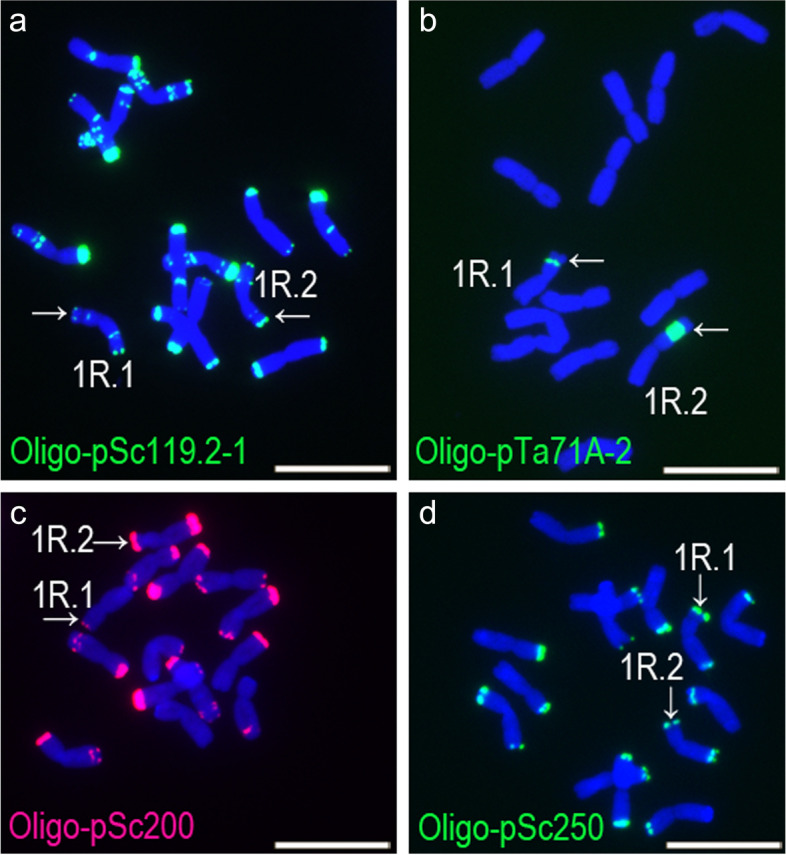
ND-FISH analysis of root-tip metaphase cells of four rye plants. **a** The two 1RS arms in a plant PI 315,959–4 with different signal intensities of Oligo-pSc119.2–1. **b** The two 1RS arms in plant PI 315,959–7 with different signal intensities of Oligo-pTa71A-2. **c** The two 1RS arms in plant PI 315,959–8 with different signal intensities of Oligo-pSc200. **d** The two 1RS arms in plant PI 315,959–5 with different signal intensities of Oligo-pSc250. The arrows indicate 1RS arms. Scale bar: 10 μm

The differential FISH signal intensity patterns of 1RS arms in the same cell may be an indicator of genetic differences between the 1RS arms because they shared the same probe concentration and image exposure times. With the exception of Oligo-TaiI, apparent differences of signal intensity were observed for 1RS arms in the same cells using probes Oligo-pSc119.2–1, Oligo-pTa71A-2, Oligo-pSc200 and Oligo-pSc250 (Fig. 5, Additional files 1, 2, 3, 4, 5, 6, 7 and 9). These differential hybridization patterns were verified in several cells of each plant. According to the signal intensity of these four probes, 41 of the 81 rye plants contained two different 1RS arms (Additional files 1, 2, 3, 4, 5, 6, 7 and 9). Differences between the two 1RS arms were noted in ten, five, 17 and nine rye plants after hybridization using probes Oligo-pSc119.2–1, Oligo-pTa71A-2, Oligo-pSc200 and Oligo-pSc250, respectively (Additional file 9). The obvious proliferation of rDNA sequences was observed in some 1RS chromosomes, for example in a plant of PI 315,959–7 (Fig. 5b). These results indicate that all of the five tandem repeats in 1RS investigated in this study displayed altered hybridization patterns, and hence they disclosed the complexity of organization of tandem repeats on 1RS and revealed new types of 1RS chromosomes in rye.

### Polymorphisms of 1BL.1RS translocation chromosomes

**Fig. 6:**
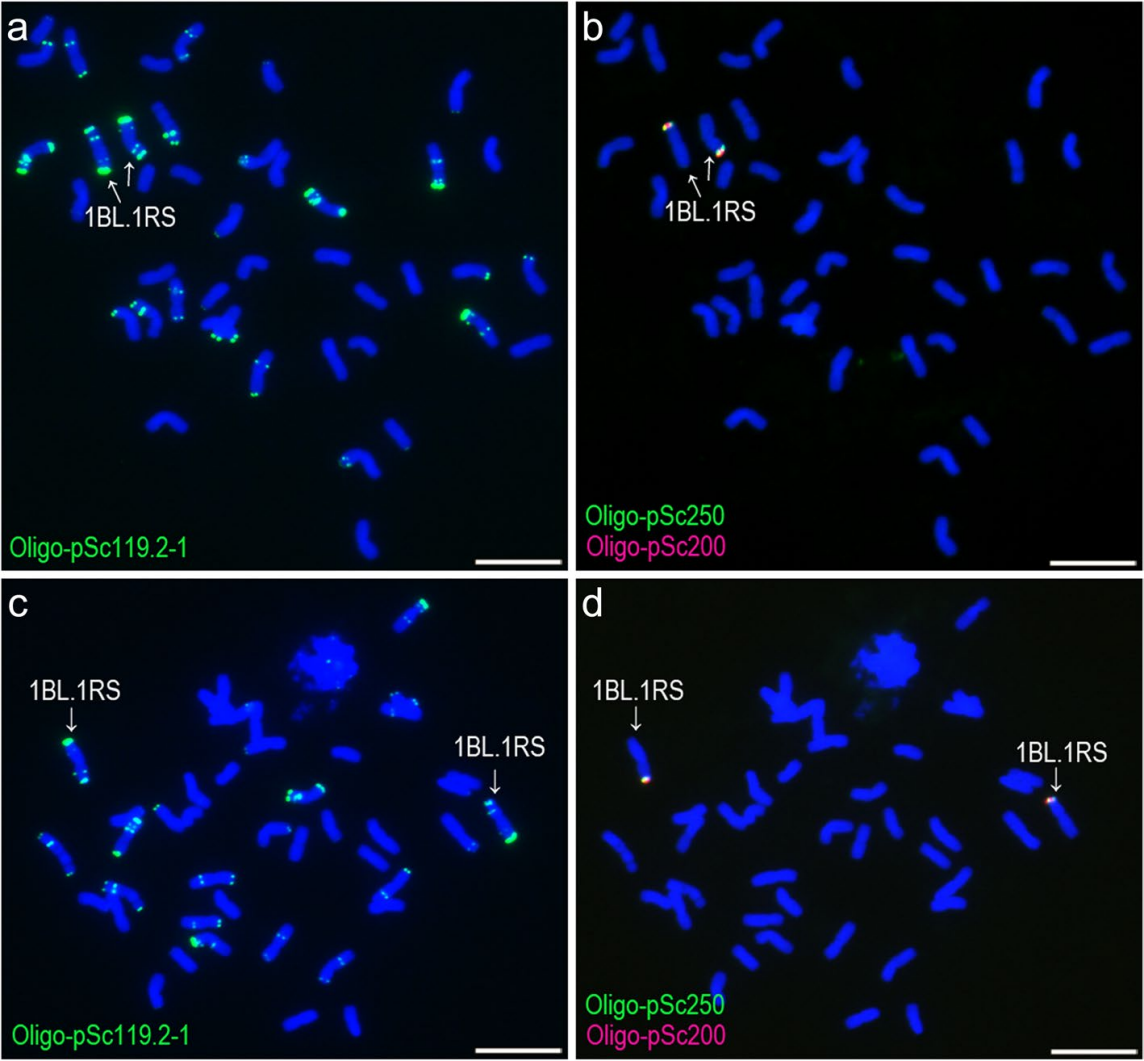
ND-FISH analysis of root-tip metaphase cells of 1BL.1RS translocations using Oligo-pSc119.2–1, Oligo-pSc200 and Oligo-pSc250. **a**, **b** A cell of Nanmai 618. **b**, **d** A cell of Wankang 58. Arrows indicate 1BL.1RS translocation chromosomes. Scale bar: 10 μm

**Fig. 7:**
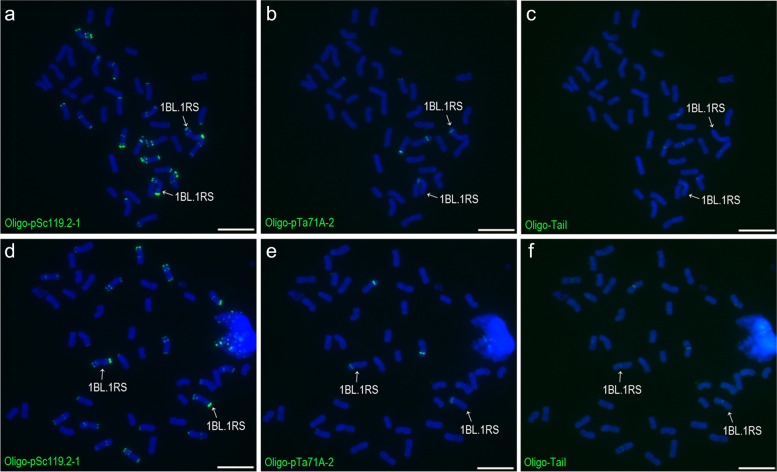
ND-FISH analysis of root-tip metaphase cells of 1BL.1RS translocations using Oligo-pSc119.2–1, Oligo-pTa71A-2 and Oligo-TaiI. **a**, **b**, **c** A cell of Nanmai 618. **d**, **e**, **f** A cell of Wankang 58. Arrows indicate 1BL.1RS translocation chromosomes. Scale bar: 10 μm

Probes Oligo-TaiI, Oligo-pSc119.2–1, Oligo-pTa71A-2, Oligo-pSc200 and Oligo-pSc250 were used to analyze 102 wheat-rye 1BL.1RS and one 1AL.1RS translocation cultivars/lines (Additional file 10). All of the 1RS arms in these cultivars/lines carried signals of Oligo-pSc119.2–1, Oligo-pTa71A-2, Oligo-pSc200 and Oligo-pSc250 (Figs. 6 and 7), and no signals of Oligo-TaiI were observed on any of these 1RS arms (Fig. 7). These results indicate that the oligo probes used in this study did not reveal any polymorphisms of the 1RS arms of these 1BL.1RS and 1AL.1RS translocations.

## Discussion

### Complexity of organization of tandem repeats in 1RS arms

Most rye cultivars have high genetic heterozygosity because they have the characteristic of high levels of cross-pollination. The heterozygosity of 1RS arms in an individual plant can be effectively detected by the FISH method using tandem repeats as probes. Tandem repeats (AAC)_5_, pSc119.2, pSc200 and pSc250 have often been used as FISH probes to investigate the karyotype polymorphism of 1R chromosomes in rye [14, 23, 24, 28, 29], but the information about the composition of tandem repeats on the 1RS arm is still limited because these tandem repeats were not used sequentially on the same chromosome spreads. Additionally, 1RS arms also contain rDNA tandem repeats and the *Tai*I family [21, 30, 31]. Cytogenetic variations of the *Tai*I family and rDNA tandem repeats on 1RS chromosomes have not been previously reported. In this study, tandem repeats pSc119.2, pSc200, pSc250, *Tai*I and rDNA were used simultaneously to investigate the polymorphisms of 1RS chromosome arms, and the three kinds of probe combinations were useful to confirm the composition of tandem repeats on individual 1RS segments.

New information about the organization of tandem repeats on 1RS chromosomes has been obtained in this study. All of the 1RS chromosome segments herein contained pSc119.2 and rDNA repeats, and variations were noted for intensities of probe signals. The obvious expansion of rDNA segments across the 1RS arms was observed but this has not been reported previously. The variations in hybridization patterns of pSc200, pSc250 and *Tai*I were exhibited by both intensity and absence of probe signals. 127 of the 162 1RS arms studied contained three kinds of subtelomeric tandem repeats, and only eight of the 1RS chromosomes contained all of the four subtelomeric tandem repeats. Most of the 1RS segments (119 of 162) contained pSc119.2, pSc200 and pSc250 sequences. Only 34 out of the 162 1RS chromosome segments contained the *Tai*I family. It has been propose that expansion of subtelomeric tandem repeats on chromosomes is caused by recombination [21, 24, 30]. The lower distribution frequency of *Tai*I than that of pSc119.2, pSc200 and pSc250 might be attributable to a lower recombination frequency in the regions containing the *Tai*I family. The multiple arrays of different tandem repeats are believed to be caused by ectopic exchanges between chromosomes [21]. Therefore, the expansion sizes of rDNA could be attributable to unequal recombination in NOR regions.

### Genetic diversity of 1BL.1RS translocation chromosome

The genetic diversity of the 1BL.1RS translocation is narrow because of the single source of the 1RS arm. In this study, the 1RS arms in commercial cultivars Lumai 14, Lovrin 10 and Aurora all originated from Petkus rye (*S. cereale* L.) [15, 32]. The 1RS segment of the 1AL.1RS translocation was derived from Insave rye (*S. cereale* L.) [33], and the 1RS in Chuannong 12 and Chuannong 17 was derived from rye line L155, which was selected from Petkus rye (*S. cereale* L.) [32, 34]. However, the originations of other 1RS arms investigated in this study are unknown according to their pedigrees (Additional file 10). The results in this study indicated that 1RS arms in Petkus rye possess extensive variations, but all of the 1RS arms in the 1BL.1RS and 1AL.1RS translocations belong to TaiI^N^-200^C^-250^C^ type, reflecting their low cytological and genetic polymorphism. Therefore, new types of 1RS should be used to enrich the diversity of 1BL.1RS translocations and importantly to replace the defeated disease resistance genes on that rye arm. Some new 1BL.1RS translocations have already been developed using different sources of 1RS [17, 28, 35–39], but the organization of tandem repeats on these 1RS arms is unclear. The results in this and previous studies [24] have discovered additional 1RS sources for creating 1BL.1RS translocations. The disease resistance reactions of all of these new 1RS chromosomes in rye is still undetermined. The 1RS arms with different cytogenetic structures should be the focus of follow-up studies and additional new allelic variations on those arms may possibly be discovered. Because the origins of most of the 1RS arms in 1BL.1RS translocations are unclear, our next plan is to integrate two different 1RS translocation chromosomes into the same plant by crossing, and to investigate their cytological characteristics based on signal intensity of probes. This may help to determine the origins of the rye segments in these 1RS translocations.

## Conclusion

The results in this study discovered the complexity of organization of tandem repeats on chromosome arm 1RS and displayed the rich polymorphism of 1RS segments in rye. New types of 1RS arms were found based on FISH hybridization signals. However, the polymorphism of 1RS arms in existing 1BL.1RS translocations was low, and it is necessary to continue to enrich the diversity of 1BL.1RS translocations for use in agriculture. The new types of 1RS found herein should be the focus of future research and their disease resistance and agronomic performances should be assessed.

## Methods

### Plant materials

A wild rye PI 531,829 (D-3249) (*Secale strictum* (C. Presl) C. Presl), six cultivated rye (*S. cereale* L.) including PI 315,959 (Petkus Kurzstroh), PI 330,965 (Petkus), PI 392,065 (Kustro), PI 446,244 (Petkus Fundulea), PI 535,171 (Petkus Ameliorant) and Chinese cv. Jingzhouheimai (Jinzhou) were used. A total of 81 seeds were randomly selected from the seven rye accessions for non-denaturing fluorescence in situ hybridization (ND-FISH) analysis. Additionally, 102 wheat-rye 1BL.1RS and one 1AL.1RS (Amigo) translocation cultivars/lines were also used to investigate the diversity of the 1RS arm (Additional file 10). Seeds of Amigo, Aurora, PI 315,959, PI 330,965, PI 392,065, PI 446,244, PI 531,829 and PI 535,171 were kindly provided by the American Germplasm Resources Information Network (GRIN). The seeds of materials 1–11 and 14–43 were kindly provided by Ennian Yang and Ling Wu (Crop Research Institute, Sichuan Academy of Agricultural Sciences, China), and Yong Ren (Mianyang Branch of National Wheat Improvement Center, Mianyang Institute of Agricultural Sciences, China). Seeds of 44–101 were kindly provided by Professor Fangpu Han (Institute of Genetics and Developmental Biology, Chinese Academy of Science, Beijing, China). The seeds of Chinese cv. Jingzhouheimai, Chuannong 12 and Chuannong 17 were from the seed store in our laboratory.

### Developing oligonucleotide probe Oligo-TaiI

Searching for tandem repeats from the IWGSC RefSeq v1.0 of Chinese Spring (http://www.wheatgenome.org) was performed according to the methods described by Tang et al*.* [40]. A tandem repeated sequence named IWGSC_RefSeq_V1_chr7B_TRF086822 was found (Additional file 11). Nucleotide BLAST search against the sequences in the Nucleotide collection (nr/nt) database in NCBI (https://blast.ncbi.nlm.nih.gov/Blast.cgi) indicated that this sequence had 91% similarity with sequence pLrTaiI-1 (GenBank Accession Number: AB016967) [25]. Therefore, the sequence IWGSC_RefSeq_V1_chr7B_TRF086822 belongs to *Tai*I family. Oligonucleotide (oligo) probe Oligo-TaiI was designed using this sequence (Additional file 11).

### Non-denaturing fluorescence in situ hybridization (ND-FISH) analysis

Oligo probes Oligo-pSc119.2–1 [26, 41], Oligo-pSc200 and Oligo-pSc250 [20, 42], Oligo-pTa71A-2 [27, 43], and Oligo-TaiI were used for ND-FISH analysis. Oligo probes were synthesized by Tsingke Biological Technology Co. Ltd. (Beijing, China), and they were 5'-end- labeled with 6-carboxyfluorescein (6-FAM), Cyanine Dye 5 (Cy5) or 6-carboxytetramethylrhodamine (TAMRA). The root-tip chromosome spreads were prepared according to the methods described by Han et al. [44]. For rye, three slides were prepared from each seed, and three kinds of probe combinations Oligo-pSc119.2–1 + Oligo-pTa71A-2 + Oligo-TaiI, Oligo-pSc119.2–1 + Oligo-pSc200 + Oligo-pSc250 and Oligo-TaiI + Oligo-pSc200 + Oligo-pSc250 were used to analyze the three slides, respectively. For each 1BL.1RS translocation cultivar/line, between three and five seeds were germinated and probe combinations Oligo-pSc119.2–1 + Oligo-pTa71A-2 + Oligo-TaiI and Oligo-pSc119.2–1 + Oligo-pSc200 + Oligo-pSc250 were used. ND-FISH analysis was carried out according to the method described by Fu et al. [42]. For each slides, at least three cells were examined.

All the methods used in this study complied with relevant institutional, national, and international guidelines and legislation.

## Supplementary Information

**Additional file 1: Figure S1.** Cut-and-paste 1R chromosomes from 11 rye plants of PI 315959. 'PI 315959-1(1R.1)' and 'PI 315959-1(1R.2)' indicate the two 1R chromosomes of the first plant of PI 315959, respectively, and so on. For each chromosome, the first three indicate hybridization with Oligo-pSc119.2, Oligo-pTa71A-2 and Oligo-TaiI, and the last three indicate hybridization with Oligo-pSc119.2, Oligo-pSc200 and Oligo-pSc250. Scale bar: 50μm.

**Additional file 2: Figure S2.** Cut-and-paste 1R chromosomes from 10 rye plants of PI 330965. 'PI 330965-1(1R.1)' and 'PI 330965-1(1R.2)' indicate the two 1R chromosomes of the first plant of PI 330965, respectively, and so on. For each chromosome, the first three indicate hybridization with Oligo-pSc119.2, Oligo-pTa71A-2 and Oligo-TaiI, and the last three indicate hybridization with Oligo-pSc119.2, Oligo-pSc200 and Oligo-pSc250. Scale bar: 50μm.

**Additional file 3: Figure S3.** Cut-and-paste 1R chromosomes from 20 rye plants of PI 392065. 'PI 392065-1(1R.1)' and 'PI 392065-1(1R.2)' indicate the two 1R chromosomes of the first plant of PI 392065, respectively, and so on. For each chromosome, the first three indicate hybridization with Oligo-pSc119.2, Oligo-pTa71A-2 and Oligo-TaiI, and the last three indicate hybridization with Oligo-pSc119.2, Oligo-pSc200 and Oligo-pSc250. Scale bar: 50μm.

**Additional file 4: Figure S4.** Cut-and-paste 1R chromosomes from 10 rye plants of PI 446244. 'PI 446244-1(1R.1)' and 'PI 446244-1(1R.2)' indicate the two 1R chromosomes of the first plant of PI 446244, respectively, and so on. For each chromosome, the first three indicate hybridization with Oligo-pSc119.2, Oligo-pTa71A-2 and Oligo-TaiI, and the last three indicate hybridization with Oligo-pSc119.2, Oligo-pSc200 and Oligo-pSc250. Scale bar: 50μm.

**Additional file 5: Figure S5.** Cut-and-paste 1R chromosomes from 10 rye plants of PI 531829. 'PI 531829-4(1R.1)' and 'PI 531829-4(1R.2)' indicate the two 1R chromosomes of the first plant of PI 531829, respectively, and so on. For each chromosome, the first three indicate hybridization with Oligo-pSc119.2, Oligo-pTa71A-2 and Oligo-TaiI, and the last three indicate hybridization with Oligo-pSc119.2, Oligo-pSc200 and Oligo-pSc250. Scale bar: 50μm.

**Additional file 6: Figure S6.** Cut-and-paste 1R chromosomes from 10 rye plants of PI 535171. 'PI 535171-1(1R.1)' and 'PI 535171-1(1R.2)' indicate the two 1R chromosomes of the first plant of PI 535171, respectively, and so on. For each chromosome, the first three indicate hybridization with Oligo-pSc119.2, Oligo-pTa71A-2 and Oligo-TaiI, and the last three indicate hybridization with Oligo-pSc119.2, Oligo-pSc200 and Oligo-pSc250. Scale bar: 50μm.

**Additional file 7: Figure S7.** Cut-and-paste 1R chromosomes from 10 rye plants of Jingzhouheimai. 'Jinzhou-1(1R.1)' and 'Jinzhou-1(1R.2)' indicate the two 1R chromosomes of the first plant of Jingzhouheimai, respectively, and so on. For each chromosome, the first three indicate hybridization with Oligo-pSc119.2, Oligo-pTa71A-2 and Oligo-TaiI, and the last three indicate hybridization with Oligo-pSc119.2, Oligo-pSc200 and Oligo-pSc250. Scale bar: 50μm.

**Additional file 8: Table S1.** 1R chromosomes derived from seven species of rye and the types of their 1RS arms.

**Additional file 9: Table S2.** Rye plants with different 1RS arms judged by signal intensity of five probes.

**Additional file 10: Table 3.** Information of materials used in this study.

**Additional file 11: Table S4.** Information of probe Oligo-TaiI.

## Data Availability

The datasets used and/or analyzed during the current study are available from the corresponding author on reasonable request.

## Abbreviations

Oligo: Oligonucleotide
ND-FISH: Non-denaturing fluorescence in situ hybridization
Jinzhou: Chinese cv. Jingzhouheimai
