# Supplementary figures and images for: Variations of subtelomeric tandem repeats and rDNA on chromosome 1RS arms in the genus Secale and 1BL.1RS translocations
Source: BMC Plant Biol. 2022 Apr 25;22:212. doi: 10.1186/s12870-022-03598-6 (PMC9036760; doi:10.1186/s12870-022-03598-6)

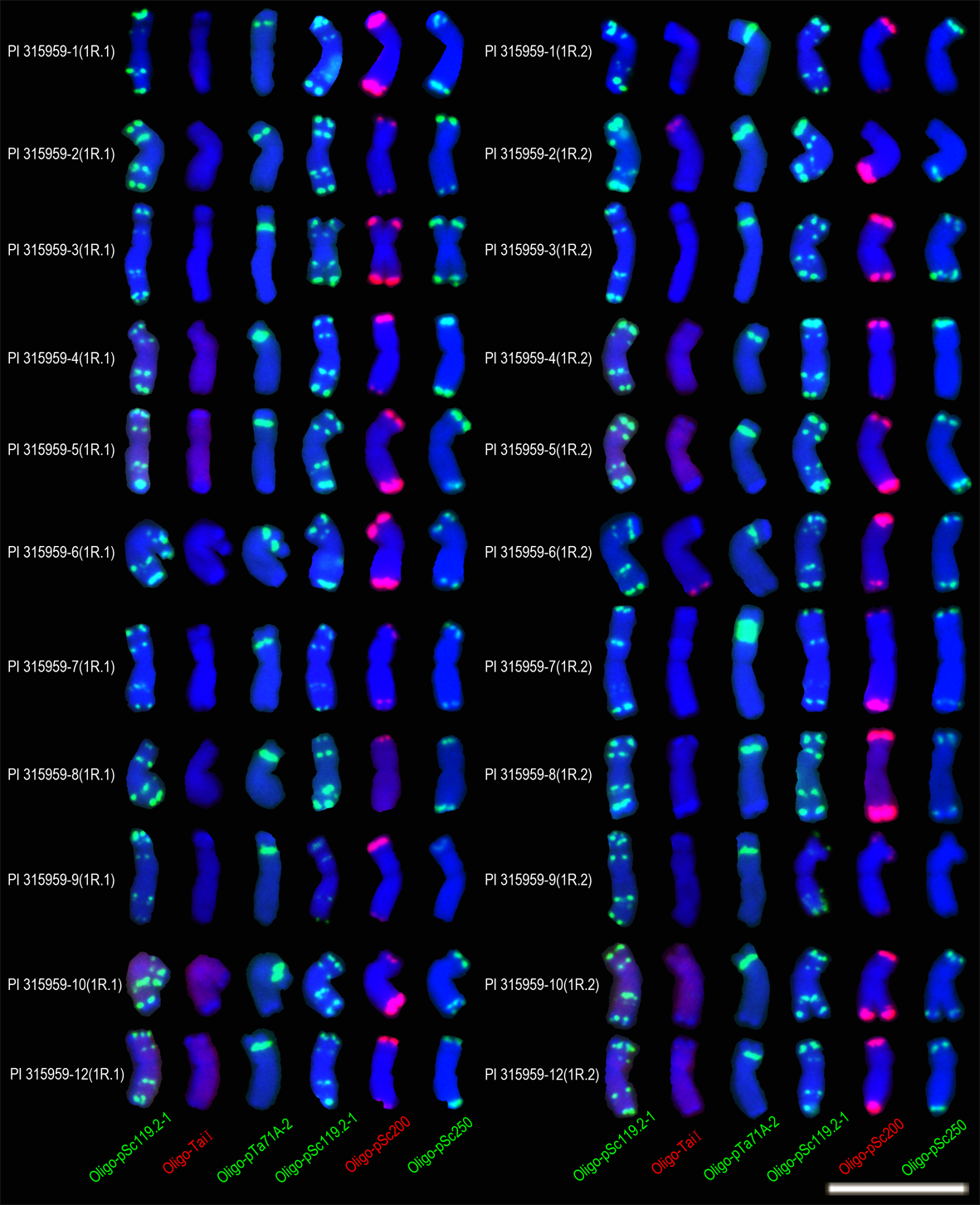

Supplement: Supplementary file 1 — Additional file 1: Figure S1. Cut-and-paste 1R chromosomes from 11 rye plants of PI 315959. 'PI 315959-1(1R.1)' and 'PI 315959-1(1R.2)' indicate the two 1R chromosomes of the first plant of PI 315959, respectively, and so on. For each chromosome, the first three indicate hybridization with Oligo-pSc119.2, Oligo-pTa71A-2 and Oligo-TaiI, and the last three indicate hybridization with Oligo-pSc119.2, Oligo-pSc200 and Oligo-pSc250. Scale bar: 50μm. [file 12870_2022_3598_MOESM1_ESM.tif]

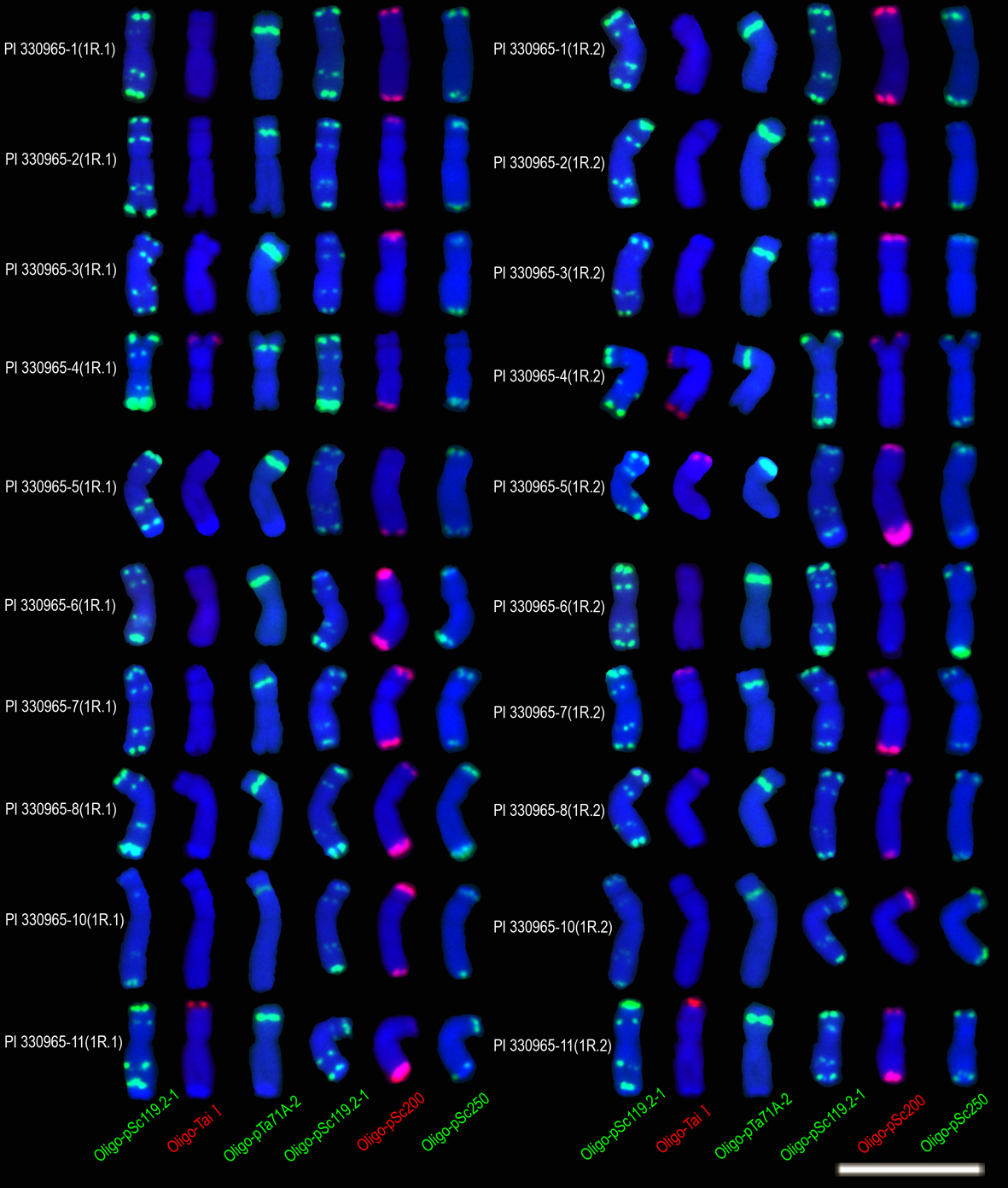

Supplement: Supplementary file 2 — Additional file 2: Figure S2. Cut-and-paste 1R chromosomes from 10 rye plants of PI 330965. 'PI 330965-1(1R.1)' and 'PI 330965-1(1R.2)' indicate the two 1R chromosomes of the first plant of PI 330965, respectively, and so on. For each chromosome, the first three indicate hybridization with Oligo-pSc119.2, Oligo-pTa71A-2 and Oligo-TaiI, and the last three indicate hybridization with Oligo-pSc119.2, Oligo-pSc200 and Oligo-pSc250. Scale bar: 50μm. [file 12870_2022_3598_MOESM2_ESM.tif]

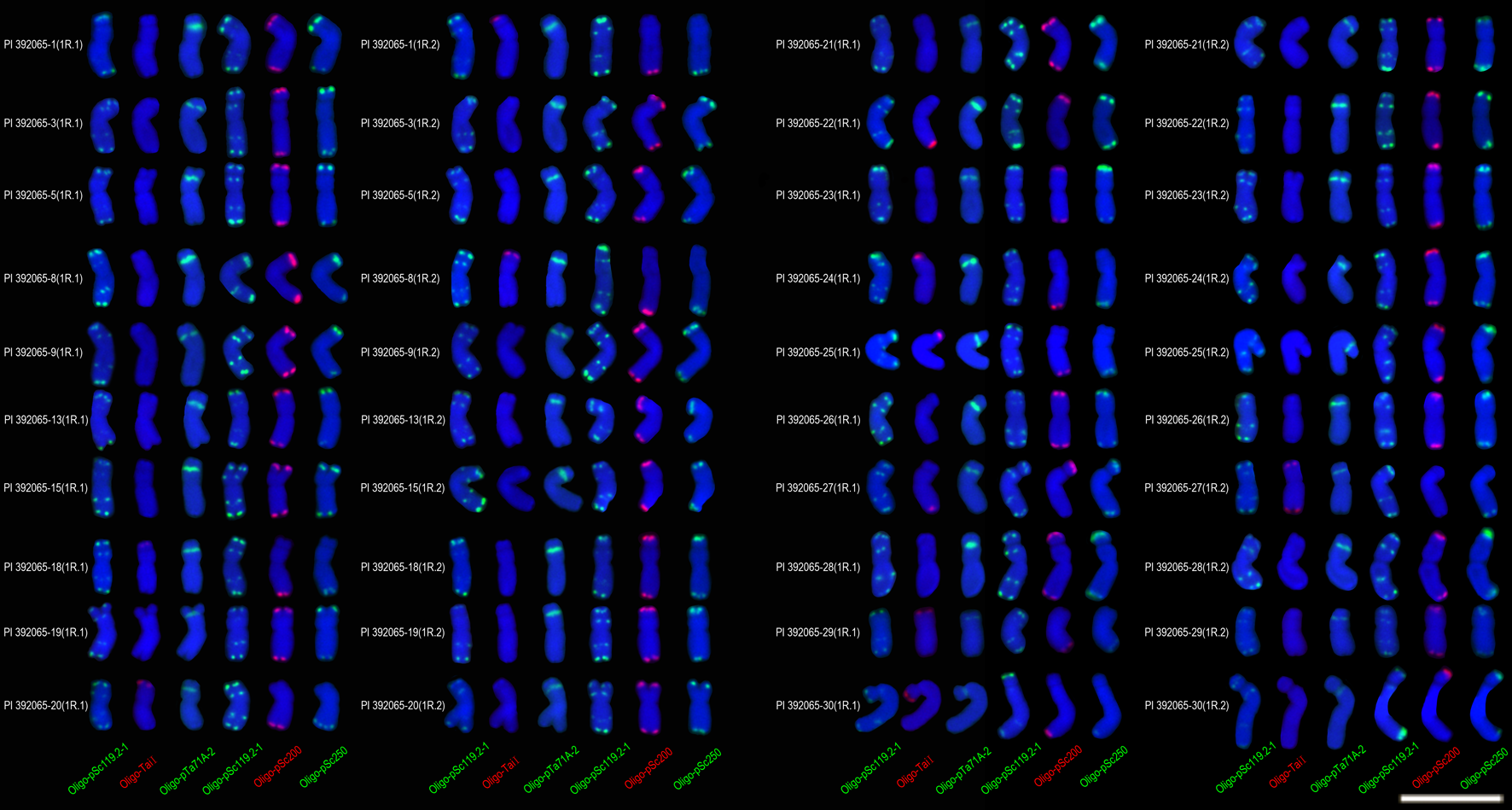

Supplement: Supplementary file 3 — Additional file 3: Figure S3. Cut-and-paste 1R chromosomes from 20 rye plants of PI 392065. 'PI 392065-1(1R.1)' and 'PI 392065-1(1R.2)' indicate the two 1R chromosomes of the first plant of PI 392065, respectively, and so on. For each chromosome, the first three indicate hybridization with Oligo-pSc119.2, Oligo-pTa71A-2 and Oligo-TaiI, and the last three indicate hybridization with Oligo-pSc119.2, Oligo-pSc200 and Oligo-pSc250. Scale bar: 50μm. [file 12870_2022_3598_MOESM3_ESM.tif]

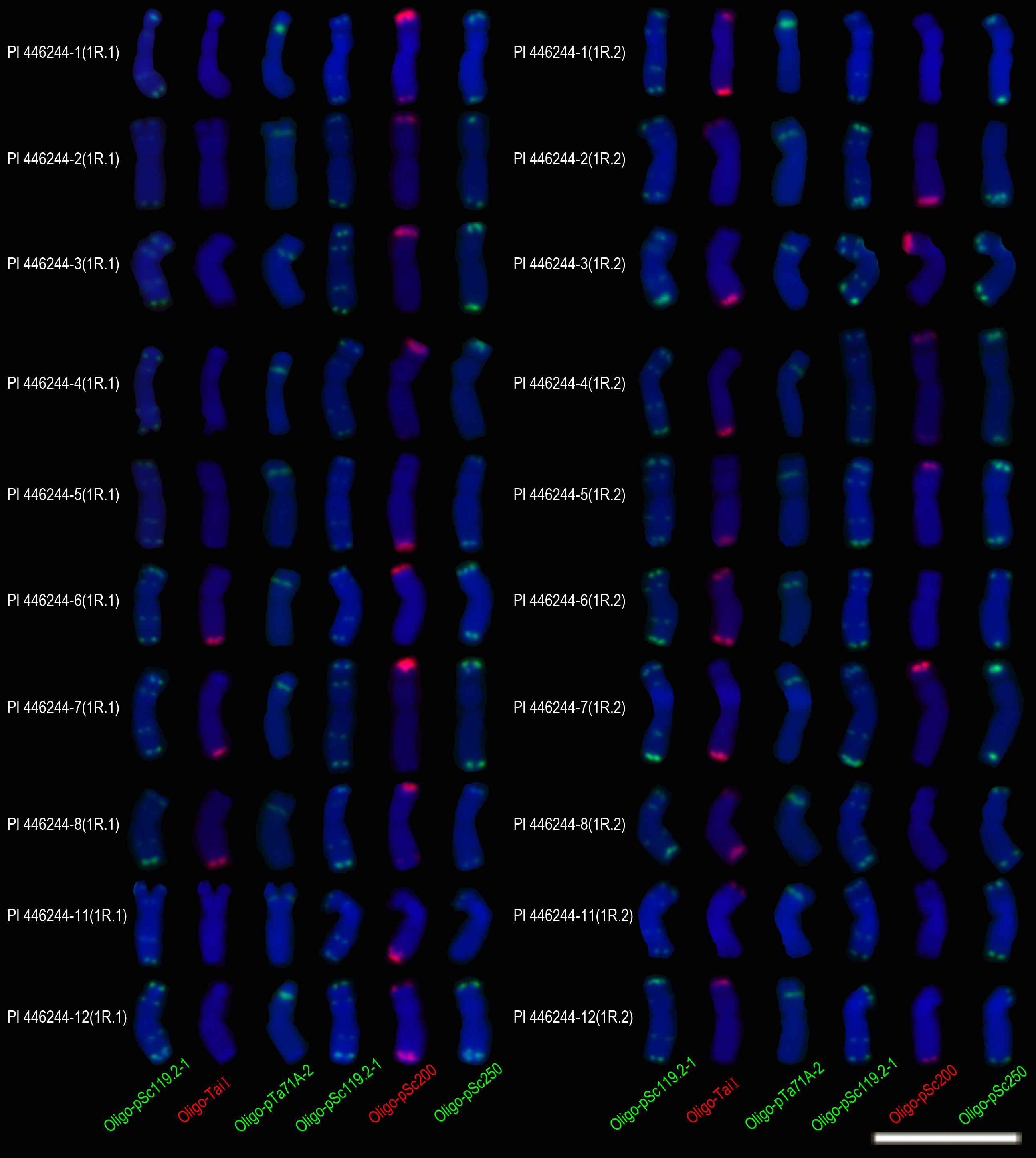

Supplement: Supplementary file 4 — Additional file 4: Figure S4. Cut-and-paste 1R chromosomes from 10 rye plants of PI 446244. 'PI 446244-1(1R.1)' and 'PI 446244-1(1R.2)' indicate the two 1R chromosomes of the first plant of PI 446244, respectively, and so on. For each chromosome, the first three indicate hybridization with Oligo-pSc119.2, Oligo-pTa71A-2 and Oligo-TaiI, and the last three indicate hybridization with Oligo-pSc119.2, Oligo-pSc200 and Oligo-pSc250. Scale bar: 50μm. [file 12870_2022_3598_MOESM4_ESM.tif]

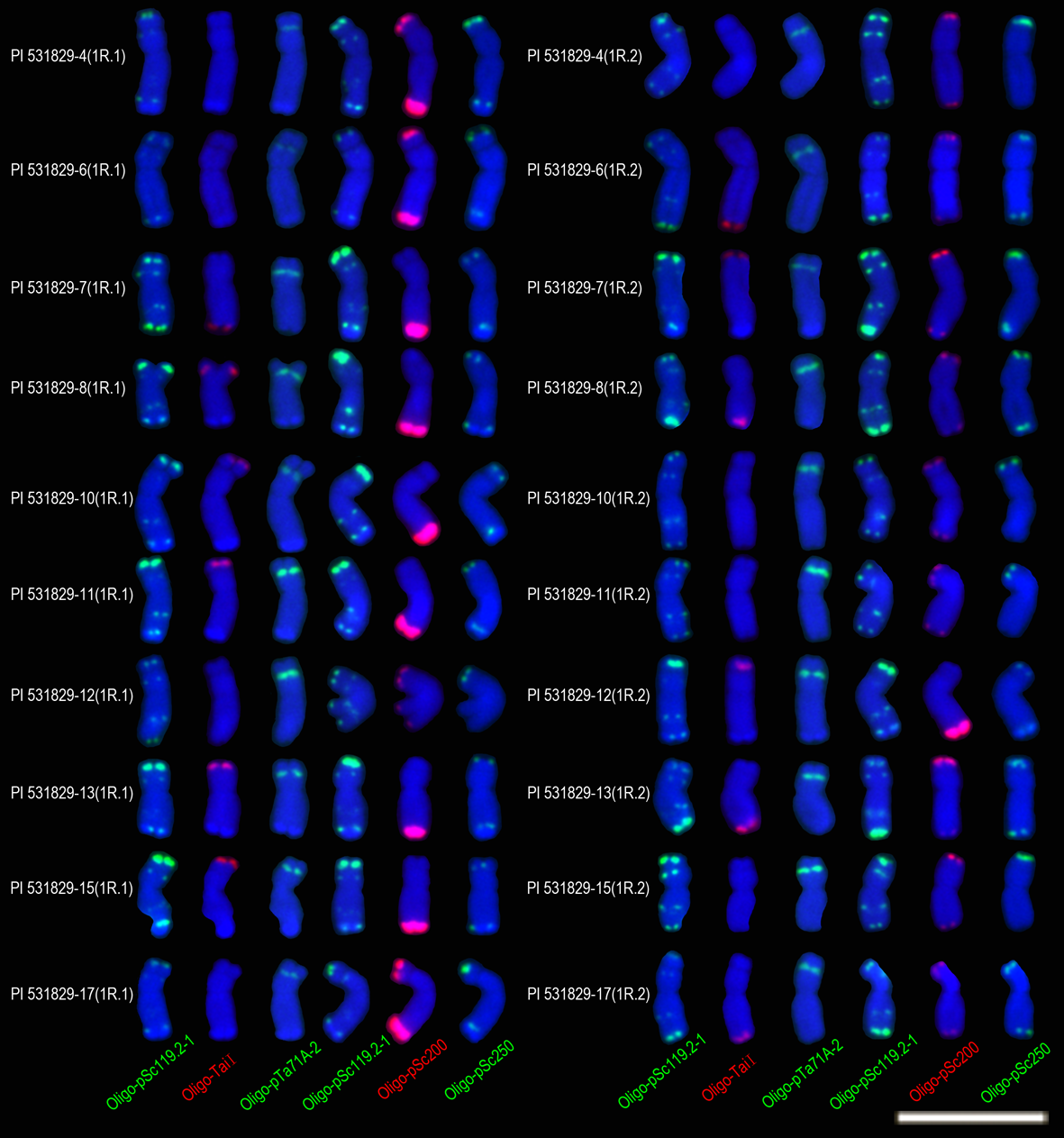

Supplement: Supplementary file 5 — Additional file 5: Figure S5. Cut-and-paste 1R chromosomes from 10 rye plants of PI 531829. 'PI 531829-4(1R.1)' and 'PI 531829-4(1R.2)' indicate the two 1R chromosomes of the first plant of PI 531829, respectively, and so on. For each chromosome, the first three indicate hybridization with Oligo-pSc119.2, Oligo-pTa71A-2 and Oligo-TaiI, and the last three indicate hybridization with Oligo-pSc119.2, Oligo-pSc200 and Oligo-pSc250. Scale bar: 50μm. [file 12870_2022_3598_MOESM5_ESM.tif]

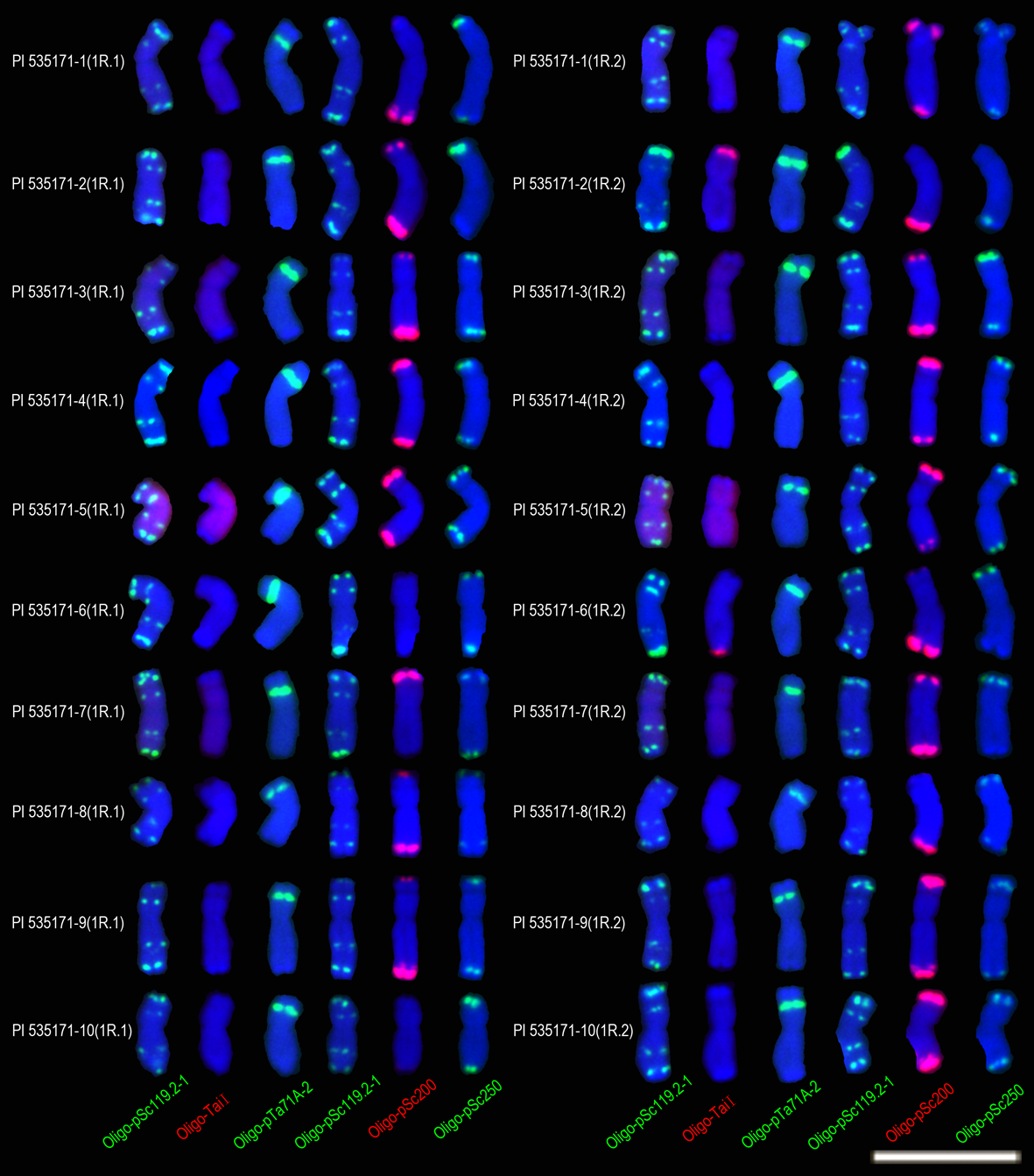

Supplement: Supplementary file 6 — Additional file 6: Figure S6. Cut-and-paste 1R chromosomes from 10 rye plants of PI 535171. 'PI 535171-1(1R.1)' and 'PI 535171-1(1R.2)' indicate the two 1R chromosomes of the first plant of PI 535171, respectively, and so on. For each chromosome, the first three indicate hybridization with Oligo-pSc119.2, Oligo-pTa71A-2 and Oligo-TaiI, and the last three indicate hybridization with Oligo-pSc119.2, Oligo-pSc200 and Oligo-pSc250. Scale bar: 50μm. [file 12870_2022_3598_MOESM6_ESM.tif]

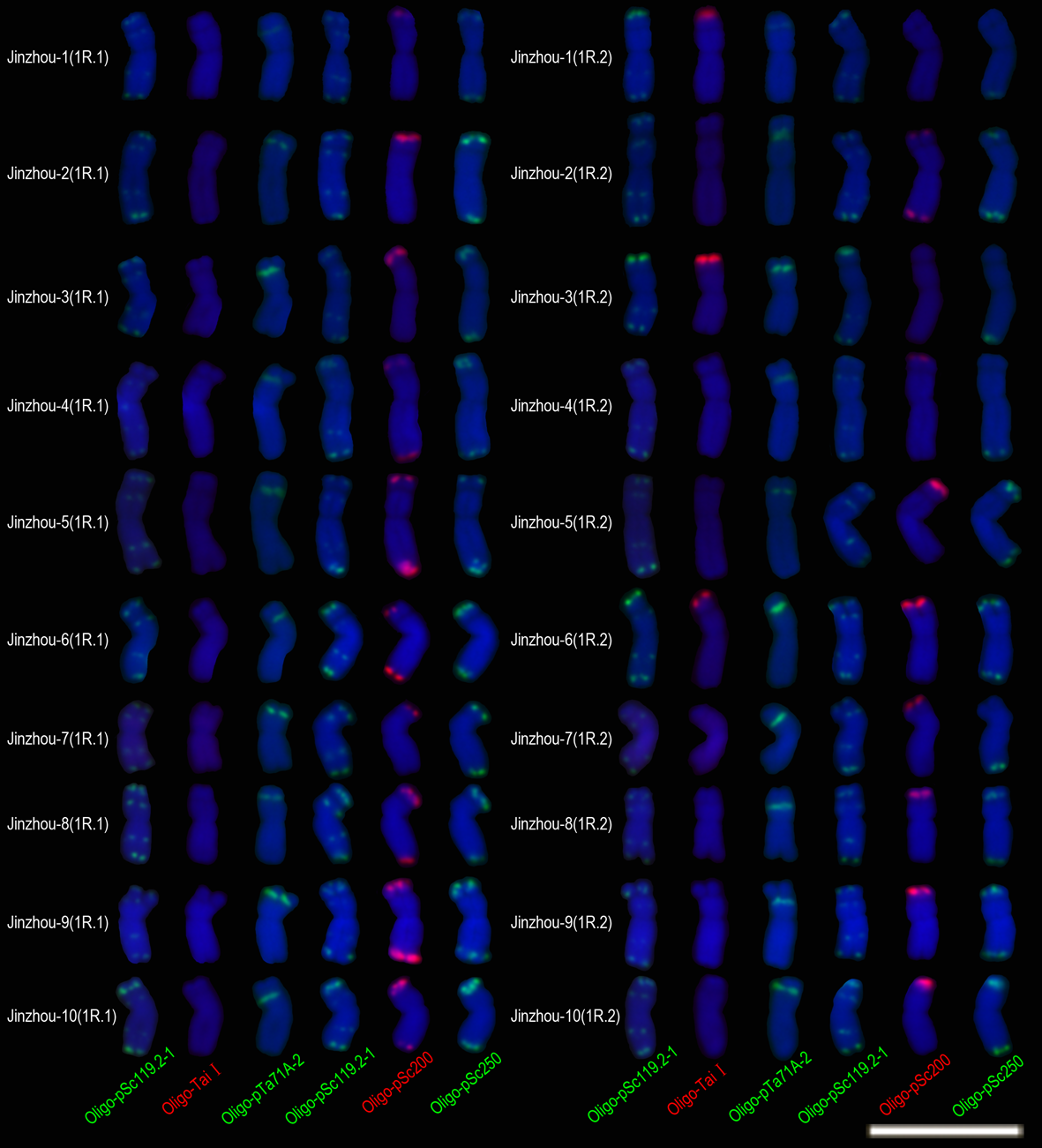

Supplement: Supplementary file 7 — Additional file 7: Figure S7. Cut-and-paste 1R chromosomes from 10 rye plants of Jingzhouheimai. 'Jinzhou-1(1R.1)' and 'Jinzhou-1(1R.2)' indicate the two 1R chromosomes of the first plant of Jingzhouheimai, respectively, and so on. For each chromosome, the first three indicate hybridization with Oligo-pSc119.2, Oligo-pTa71A-2 and Oligo-TaiI, and the last three indicate hybridization with Oligo-pSc119.2, Oligo-pSc200 and Oligo-pSc250. Scale bar: 50μm. [file 12870_2022_3598_MOESM7_ESM.tif]
