# Supplementary material for: Variations of subtelomeric tandem repeats and rDNA on chromosome 1RS arms in the genus Secale and 1BL.1RS translocations
Source: BMC Plant Biol. 2022 Apr 25;22:212. doi: 10.1186/s12870-022-03598-6 (PMC9036760; doi:10.1186/s12870-022-03598-6)
Supplement: Supplementary file 9 — Additional file 9: Table S2. Rye plants with different 1RS arms judged by signal intensity of five probes. [file 12870_2022_3598_MOESM9_ESM.docx]

Tbale 2. Rye plants with different 1RS arms judged by signal intensity of five probes *.

| Probe | Oligo-pSc119.2-1 | Oligo-pTa71A-2 | Oligo-TaiI | Oligo-pSc200 | Oligo-pSc250 |
| --- | --- | --- | --- | --- | --- |
| Plants with apparent difference in signal intensity between two 1RS arms | PI 315959-4  PI 330965-6  PI 531829-8  PI 531829-10  PI 531829-11  PI 531829-12  PI 531829-13  PI 535171-2  PI 535171-3  Jinzhou-3 | PI 315959-1  PI 315959-2  PI 315959-7  PI 330965-2  PI 392065-28 | - | PI 315959-1  PI 315959-8  PI 315959-9  PI 315959-10  PI 330965-1  PI 330965-5  PI 330965-6  PI 392065-21  PI 392065-23  PI 446244-7  PI 531829-4  PI 531829-6  PI 531829-17  PI 535171-9  Jinzhou-2  Jinzhou-5  Jinzhou-6 | PI 315959-5  PI 392065-21  PI 392065-23  PI 531829-7  PI 535171-1  PI 535171-3  PI 535171-10  Jinzhou-2  Jinzhou-5 |

*The plants with different 1RS arms displayed by presence and absence of signals are not listed in this Table. "-" indicates no plants containing both 1RS arms with signal of Oligo-TaiI.
