# Supplementary material for: Variations of subtelomeric tandem repeats and rDNA on chromosome 1RS arms in the genus Secale and 1BL.1RS translocations
Source: BMC Plant Biol. 2022 Apr 25;22:212. doi: 10.1186/s12870-022-03598-6 (PMC9036760; doi:10.1186/s12870-022-03598-6)
Supplement: Supplementary file 11 — Additional file 11: Table S4. Information of probe Oligo-TaiI. [file 12870_2022_3598_MOESM11_ESM.docx]

Table S4. Information of probe Oligo-TaiI

| Probe | Probe sequences | Dosage used for each slide (ng/slide) | Original sequences used for designing probes |
| --- | --- | --- | --- |
| Oligo-TaiI | GCCCCCCTGGTAGGGACAAACGTACCTTTCTGCAGATGCTCTATGATCCGGGC | 30.0 | CTTATAGACATTAGCCCCCCTGGTAGGGACAAACGTACCTTTCTGCAGATGCTCTATGATCCGGGCAATCTCATTTGGCCGTAAATTGAAGAAAATCCAGCGGCTATATGCCTTATTCTCCTTGTTTTCGCCGGATGGCATGCTGTAACTCACGCGCGTGAAAGCCAAGAACAAAAGGGCATTCCTGGCATCGGTCAATATATATGTGGAAACGATATCTCCTCTATAGAGGACAAGAAATTTTGGGGCAATCAGGCAAAAAGCGCACGAGAAAATGAAGTACTTTCAAGGACTGCCATAGAATGGAGAGATGTTCACTAAAACGGTCATAATTTTGTCATTCGGAGTCCGTGTCGGACCCACAAGTACTCAAACTGCTCGCGGCGAGCGCGCGAATCTAGAAAATACTCCCCGCTTGCGCCAGGAGGCCCGGAGTGCCCTCAAATACGGCCTTAAGCAGGACTTTTTGGCCCTGAAAGTCAAAGAACATCATTCGTCGCTGTTTTTCAGACAAGTTTCTGAACCCATTGGCACTTCCTTTTGAAGGAAATTCGAGAGATTATGTTT |
